# An exploratory study of electronic medical record implementation and recordkeeping culture: the case of hospitals in Indonesia

**DOI:** 10.1186/s12913-025-12399-0

**Published:** 2025-02-14

**Authors:** Md. Khalid Hossain, Juliana Sutanto, Putu Wuri Handayani, Anasthasia Agnes Haryanto, Joy Bhowmik, Viviane Frings-Hessami

**Affiliations:** 1 Department of Human Centred Computing, Monash University, Melbourne, VIC Australia; 2 Faculty of Computer Science, University of Indonesia, Depok, West Java Indonesia; 3 Center for Sustainable Development, University of Liberal Arts Bangladesh, Dhaka, Bangladesh

**Keywords:** Electronic medical record, Recordkeeping culture, Indonesia, Hospitals, Regulation, Information

## Abstract

**Background:**

The digitization of healthcare, through electronic medical records (EMRs), is recognized globally as a transformative initiative. Indonesia mandated all healthcare facilities to adopt EMRs by December 31, 2023. However, this transition is complicated by diverse technological, cultural, and infrastructural challenges, with little research addressing the recordkeeping culture's impact on EMR adoption. This study investigates the electronic recordkeeping culture in Indonesian hospitals following a government mandate to adopt Electronic Medical Records (EMRs). It aims to understand the readiness and challenges in implementing EMRs across hospitals on Java and Sulawesi islands, focusing on infrastructure, staff digital skills, and varied adoption approaches.

**Methods:**

A qualitative case study approach was utilized, involving focus groups and semi-structured interviews with 150 staff from 12 hospitals. Conducted between November 2023 and June 2024, the study applied thematic analysis based on Oliver and Foscarini’s (2020) recordkeeping culture framework to explore organizational readiness, technological infrastructure, and healthcare professionals' skills in managing electronic records.

**Results:**

The findings reveal significant differences in EMR adoption between the islands. Hospitals on Java exhibited proactive engagement, supported by better technological infrastructure and staff training programs, while Sulawesi hospitals adopted EMRs primarily to meet regulatory requirements. Challenges included inconsistent internet connectivity, low digital literacy among staff, and ongoing reliance on paper records during the transition. Some Java hospitals have begun fostering a culture conducive to electronic recordkeeping by focusing on developing staff skills in EMR management.

**Conclusions:**

The effectiveness of EMR adoption in Indonesia relies on addressing technological infrastructure issues and enhancing staff digital literacy. While progress has been made, particularly in more developed regions, a cohesive national strategy emphasizing technological support and targeted training is essential to fully realize the benefits of EMRs in improving healthcare and recordkeeping standards across Indonesia.

**Supplementary Information:**

The online version contains supplementary material available at 10.1186/s12913-025-12399-0.

## Introduction

The digitization of healthcare systems is increasingly becoming a global priority, with electronic medical records (EMRs) playing a pivotal role in this transformation [1, 2]. EMRs play a critical role in healthcare by providing a holistic view of a patient's health, thereby enabling clinicians to make informed decisions with confidence and to share information effectively across patient care teams [3]. The adoption of EMRs in medical practice has notably increased in recent years, offering several advantages such as rapid and remote access to patient data [4], improved administrative efficiency [5], enhanced legibility and accuracy of data [6], and a reduction in medical errors [7]. EMR represents a comprehensive system rather than just a standalone tool, serving as a critical asset in managing patients’ medical histories. It encompasses an array of computerized clinical information systems designed to capture, store, and present patients’ data [5, 8]. The multifaceted nature of EMR encompasses a clinical data repository, clinical decision support, controlled medical vocabulary, order entry, computerized provider order entry, pharmacy, and clinical documentation applications [9].

Over the past two decades, a significant body of academic research has emerged, focusing on the implementation of EMR systems. These studies explored various facets of the EMR adoption process, including technological challenges, financial constraints, user resistance, data security, and interoperability issues [8–13]. Most of these studies aim to shed light on the technical and operational hurdles that healthcare providers face when transitioning from paper-based systems to digital solutions. However, much of this research is based on the experiences of developed countries, where the necessary infrastructure for EMR adoption is more advanced and the cultural context is more uniform. While these studies provide valuable insights, one critical aspect that remains largely overlooked is the recordkeeping culture of the hospitals that are expected to adopt and integrate EMRs. Recordkeeping culture refers to the collective attitudes, practices, beliefs, and norms surrounding how records are created, maintained, stored, and used within an organization [14, 15]. This culture significantly influences how successfully an EMR system is implemented and integrated into the daily operations of hospitals. Understanding recordkeeping culture is vital because it shapes how information is documented, accessed, and utilized in healthcare settings, which in turn impacts the quality of patient care.

Hospitals, as complex organizations, have long relied on paper-based records, which come with their own established practices, habits, and cultural norms [16, 17]. Transitioning to an electronic system is not just a technological change—it is also a profound cultural shift in the way information is managed and utilized [18]. However, most of the current academic research on EMR implementation fails to address this cultural dimension, focusing instead on technical and organizational issues [8–13]. The absence of attention to recordkeeping culture is especially problematic in countries like Indonesia, where hospitals vary significantly in terms of size, administrative structures, levels of technological development, and familiarity with digital systems [19, 20]. This paper seeks to address this gap in the literature by using the recordkeeping culture framework developed by Oliver and Foscarini [14]. Their framework provides a comprehensive lens through which to examine how organizational cultures shape recordkeeping practices, including the creation, maintenance, and use of records. Applying this framework to the case of hospitals in Indonesia allows for a more nuanced understanding of the challenges and opportunities involved in the transition from paper-based to electronic records.

Indonesia’s healthcare system presents a unique context for exploring the intersection of EMR implementation and recordkeeping culture. The country has a diverse and complex healthcare system [21], with thousands of public and private hospitals ranging from large urban medical centers to small rural clinics [22, 23]. These hospitals operate under different administrative systems, have varying access to technology, and employ healthcare professionals with diverse educational and cultural backgrounds [19, 20]. In recent years, Indonesia’s healthcare system has undergone significant reforms, most notably the introduction of the national health insurance scheme [21, 24]. This reform has increased the demand for healthcare services [25], placing additional pressure on healthcare institutions to adopt more efficient and scalable systems of recordkeeping. EMRs have been identified as a key component of this modernization process as the Government of Indonesia mandated the transition from physical to electronic medical records for all healthcare facilities By December 31, 2023 [26]. However, their implementation has been inconsistent across the country, with larger, better-funded hospitals in urban areas more likely to adopt EMR systems, while smaller, rural hospitals may lag behind [27, 28].

Given this diversity, understanding the recordkeeping culture of hospitals in Indonesia is essential for identifying the underlying factors that either facilitate or impede the successful implementation of EMRs. Although previous research on EMR implementation in Indonesia has focused on technical challenges, such as the lack of financial resources, inadequate IT infrastructure, limited staff training, and concerns over data privacy and security [27–30], it has largely neglected the cultural factors at play. These studies often provide a limited view of the reasons why some hospitals succeed in implementing EMRs while others struggle. In this regard, Oliver and Foscarini’s [14] recordkeeping culture framework is particularly useful for exploring these cultural dimensions. Their framework emphasizes that recordkeeping is not merely a technical process but a deeply ingrained organizational practice shaped by institutional values, professional norms, legal and regulatory requirements, and the attitudes of employees [14]. Applying this framework allows for a more holistic examination of the factors influencing EMR adoption, moving beyond technical and organizational concerns to include the cultural underpinnings of recordkeeping in Indonesian hospitals.

For many Indonesian hospitals, especially those in rural areas, paper-based recordkeeping has been the dominant practice for decades [31]. This has led to the development of deeply entrenched habits and routines, which can be difficult to change even in the face of evidence demonstrating the benefits of EMRs [3–7]. Cultural resistance to change is often a significant barrier to EMR adoption [32]. Studies from other countries have shown that healthcare professionals may resist the transition to EMRs because they are accustomed to paper-based systems and perceive new technologies as complicated or time-consuming [11, 33, 34]. The shift from paper to electronic records requires not only technical training but also a broader cultural shift in how healthcare professionals think about and interact with patient data [18, 35]. The recordkeeping culture framework proposed by Oliver and Foscarini [14] provides a valuable tool for understanding these cultural dynamics, as it highlights the importance of organizational attitudes and practices in shaping the success of recordkeeping systems. By applying this framework, this study aims to identify the key cultural factors that facilitate or hinder the adoption of EMRs in Indonesian hospitals.

This research is exploratory in nature, given the lack of prior studies on the recordkeeping culture of Indonesian hospitals and its impact on EMR implementation. The study involved qualitative interviews and focus groups with healthcare professionals, hospital administrators, and IT staff across a range of hospitals in Indonesia from distinct geographic locations with significant differences in urbanization and development statuses. These interviews and focus groups provided insights into the recordkeeping practices of these hospitals and how they are influenced by different factors. Ultimately, this study aims to contribute to the academic literature on EMR implementation by highlighting the role of recordkeeping culture in shaping the adoption process. By focusing on the case of hospitals in Indonesia, the research seeks to provide practical insights for policymakers, healthcare administrators, and IT professionals involved in the implementation of EMRs for fostering a supportive recordkeeping culture that facilitates the successful transition to electronic records in Indonesian hospitals.

### Records and electronic medical recordkeeping

According to the ISO 15489 a record is information generated, received, and preserved as evidence by an organization or individual, either to fulfill legal requirements or conduct business activities [36]. In line with this definition, electronic medical records are patient’s healthcare information recorded digitally or converted from paper records with a combination of text, graphics, data, audio, pictorial, or other digitally represented information that are created, modified, maintained, archived, retrieved, or distributed by a computer system [37].

The existing body of literature on EMR systems encompasses a wide array of research, including investigations into their implementation across various countries and healthcare settings. For instance, Zarcadoolas et al. [38] conducted a study in the USA, focusing on the perceptions of vulnerable consumer groups, such as individuals with low education levels and racial or ethnic minorities, towards patient portals within EMR systems. Their findings underscored the importance of designing patient portals that are accessible, visually appealing, and user-friendly to cater to the needs of these consumer groups. Furthermore, Baltaxe et al. [39] examined the implementation of EMR in Integrated Critical Care (ICC) programs in Europe, revealing that while partial implementation has been achieved, comprehensive plans for future mature implementation are underway. This study also shed light on the absence of well-defined macro-level digital transformation policies and effective operational implementation plans within the healthcare systems where ICC programs are situated.

In the context of developing economies, Derecho et al. [40] identified key enablers and challenges associated with EMR adoption. Their systematic review emphasised factors such as computer literacy, education, voluntariness, and system functionality as enablers while highlighting challenges related to training, resistance to change, limited time for learning complex systems, and the costs of technology adoption. Additionally, Mwogosi and Mambile [41] identified determinants like perceived usefulness, support and training, interoperability, data security, business culture, and leadership as critical factors influencing the adoption of electronic health record systems (EHRS) among healthcare employees in Tanzania. Moreover, Bhanushali et al. [42] explored the determinants impacting EMR adoption among healthcare professionals in India, revealing the significant influence of factors such as software costs, time availability, technical competence of existing manpower, and percentage footfall. Scholl et al. [43] explored EMR adoption challenges in Indian hospitals, highlighting resistance from personnel lacking IT skills and strategies to overcome these barriers through institutional support.

Furthermore, Akwaowo et al. [44] conducted a study on the adoption of EMR in Nigeria, utilizing Davis’s technology adoption model to identify contextual and situational factors that act as barriers to EMR adoption in developing countries. Their findings underscored the importance of awareness, training, and education in increasing EMR adoption. In addition to these studies, a plethora of research has delved into various aspects of EMR adoption as highlighted in the previous section, covering topics such as the feasibility of collaborative EMR systems dealing with structured and unstructured data [45], physicians’ willingness [46] and factors influencing them to adopt EMR [47], readiness for EMR implementation [48], interoperability of EMR systems [49], improve the usability and usefulness of EMR systems considering their roles as communication support tools with the users’ wants and priorities [50], and barriers to EHR adoption in different healthcare settings.

Based on the review of existing literature on EMR adoption, it is found that most of the available studies discuss EMRs from a general perspective without relating to the theoretical background of recordkeeping issues. For example, Kim et al. [51] explored what information is valuable to doctors when they want to access medical records using mobile devices, finding that inpatient lists, lab results, and investigation lists were key. Although they did not discuss it from the perspective of recordkeeping, Oliver and Foscarini [14] mentioned the value accorded to information while defining the recordkeeping culture, which is the core conceptual framework of this paper. Dainton and Chu [52] mentioned “record keeping” by mobile medical service trips (MSTs) without discussing it based on recordkeeping processes. Similarly, Tough and Lihoma [53] referred to medical “record keeping” systems in Malawi but the findings in their research lacked a recordkeeping perspective. Several other researchers also mentioned “record keeping” in their EMR studies [54–58], yet none considered relevant standards and the background of recordkeeping and associated culture.

In the existing literature, the term “record keeping” is frequently used as two separate words, implying a traditional, physical approach to managing records. This approach is typically associated with records managers and archivists handling paper-based records [59]. In contrast, the present study adopts the single-word term “recordkeeping”, which reflects the modern management and processing of recorded information through contemporary communication and information technologies [59]. Recordkeeping as the single-word description used in the paper mostly to discuss about the recordkeeping culture refers to the comprehensive processes by which individuals create, capture, organize, and pluralize records [59]. It encompasses the management and processing of recorded information generated by agents—typically people or communication apparatuses—during their activities, as well as the establishment of recordkeeping systems prior to the creation of records to meet the needs of various stakeholders. This includes capturing, archiving, and disseminating information as evidence through modern communication and information technologies [59], making the concept relevant for electronic medical recordkeeping and associate recordkeeping culture.

### Recordkeeping culture

**Fig. 1:**
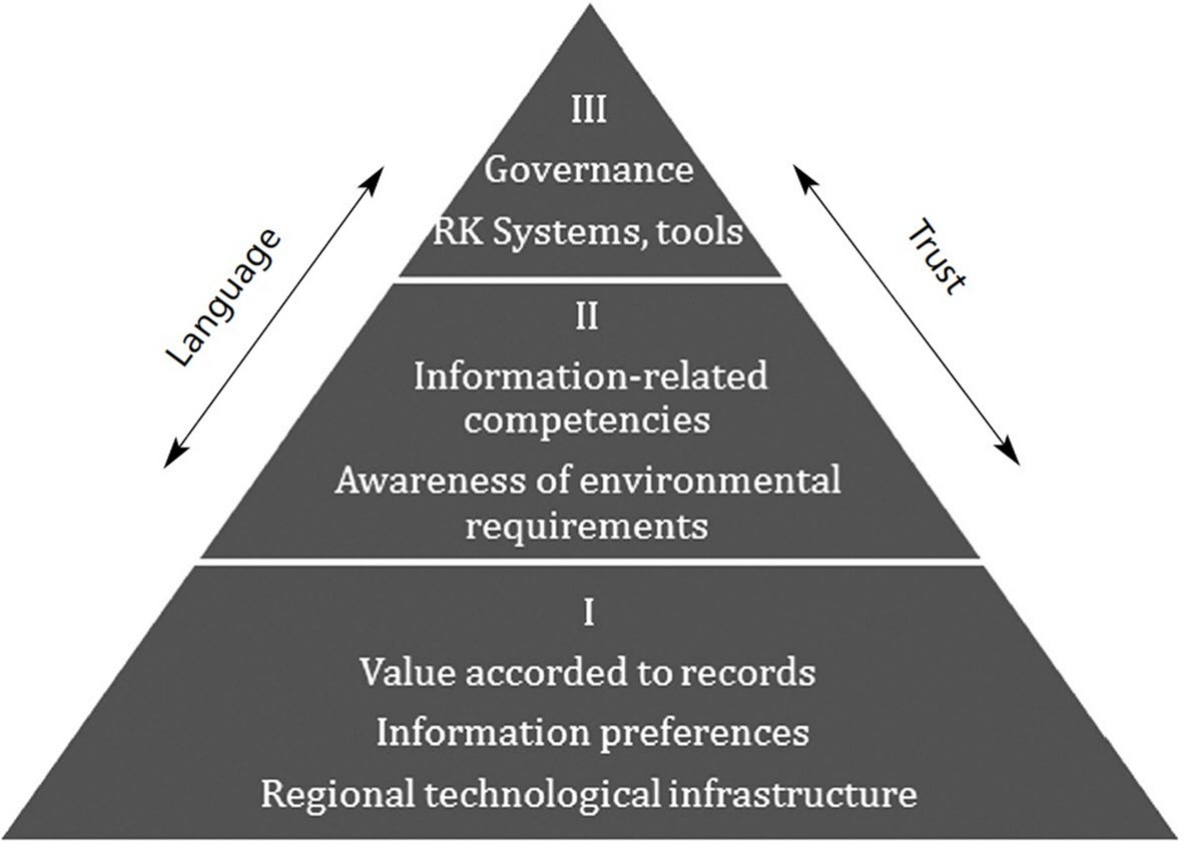
Information culture framework [14]

In 2014, Oliver and Foscarini [14] proposed a three-level information culture framework as shown in Fig. 1, which they later refined to emphasize information objects that represent present and past actions for accountability purposes, coining this as “recordkeeping culture”, which can be used to assess and analyze the cultural aspects of recordkeeping practices in organizations.

The framework recognizes that recordkeeping practices are not only shaped by policies and technology but also by the cultural context in which they are situated. It emphasizes that understanding people’s values and attitudes towards records is crucial, as humans are the primary drivers in this process. This three-level framework provides a useful structure for analyzing recordkeeping cultures, allowing organizations to identify gaps between formal policies and actual behaviors, and to develop strategies for improving their recordkeeping practices in alignment with their organizational culture. Since recordkeeping extends beyond those directly responsible for managing records and involves various professionals, especially where organizational activities are recorded in multiple business systems, ensuring proper recordkeeping within an organization requires harmony among these three levels. For example, electronic medical records necessitate the collaboration of doctors, surgeons, nurses, pathology staff, hospital administrators, IT professionals and health insurance personnel. This underscores the need for all professionals to work collaboratively, facilitated by computerized systems, signifying those computers, individuals, and organization collectively process information [2–5].

The first level of the framework comprises three factors: the value accorded to records, indicating people’s recognition and awareness of managing certain information for accountability purposes; preferences for communication formats and media; and the regional technological infrastructure where the organization is located. The first level of the framework is the foundation level as it refers to the underlying, often unconscious beliefs and assumptions that influence how people in an organization perceive the importance and role of records. These assumptions are harder to identify because they are taken for granted, but they shape the organization’s approach to recordkeeping in profound ways.

The second level represents the skills, knowledge, and expertise of people in recordkeeping. It is vital to comprehend the capacities of those managing records, including their awareness of societal and organizational requirements, such as local laws on data privacy and cybersecurity. Building on the foundational influences at the bottom layer, the middle layer identifies issues necessary for establishing proper recordkeeping systems within the organization. This includes determining if staff require training to enhance digital literacy or knowledge of relevant legislation and organizational requirements [14].

The tip of the pyramid-shaped framework has two interlinked features crucial for successful recordkeeping: the organization’s information governance and its recordkeeping systems and tools. Therefore, this level includes the visible and tangible aspects of recordkeeping culture, such as organizational structures, policies, procedures, and technologies that support recordkeeping. It is the most easily observed level of recordkeeping culture, as it involves the explicit manifestations of how records are created, managed, and maintained.

Previous studies have utilized the framework or its components in various contexts. For instance, Lian and Oliver [60] applied the framework to explore information culture in Mainland China, identifying several cultural factors influencing organizational information culture, such as egocentrism, guanxi (relationships), *mianzi* (face), *hexie* (harmony), and *renqing* (mutual benefits). In another study, a significant metalevel of the information culture framework, language and communication was studied by Frings-Hessami and Oliver [61] in an international aid organization to understand cultural clashes in information systems. They discovered that a lack of understanding of local communication preferences resulted in employees not using the information systems as intended. Lian et al. [62] previously applied the framework to investigate information culture and recordkeeping in two Chinese companies, highlighting that inadequate recordkeeping and information management systems can lead to the loss of valuable records when staff leave with information stored on personal devices. To ensure the long-term preservation of company records, systematic regulations and integrated recordkeeping functions are essential. Additionally, Frings-Hessami et al. [63] emphasized the importance of developing a recordkeeping culture in community-based organizations to encourage better recordkeeping practices, thereby enhancing the effectiveness and sustainability of these organizations. Based on these studies, we found that recordkeeping culture would be useful in this study to determine peoples’ respect towards medical records, their attitudes towards creating, organizing, and sharing records, and the requirements for electronic medical recordkeeping systems for successful long-term preservation in the hospitals in Indonesia.

## Methods

### Case study research design

As mentioned before, to realize the research aims, we used a qualitative research method by following a case study research design using qualitative data collection techniques [64] with focus groups and face-to-face semi-structured interviews with hospital staff. Due to its usefulness in exploring any new phenomenon [65], we used the qualitative research method to understand the recordkeeping cultures of hospitals in Indonesia by observing their EMR implementation practices and approaches. From November 2023 to June 2024, we collected and analyzed interview data to understand different dimensions of recordkeeping cultures in line with the recordkeeping culture framework proposed by Oliver and Foscarini [14], like the organizational perspectives, capacities, and practices related to electronic medical recordkeeping. The framework is a relatively new theoretical tool that has been increasingly applied in various organizational contexts [60–62] to examine recordkeeping practices. While it has not undergone formal validation in all settings, its growing use in academic studies highlights its relevance and utility. For our study, we intentionally did not modify the framework, as our aim was to explore its applicability in capturing the electronic medical recordkeeping culture in Indonesian hospitals. This approach was guided by the absence of alternative frameworks specifically addressing recordkeeping culture and allowed us to evaluate its effectiveness in this novel context without introducing additional variables. However, we contextualized all seven features of the framework for the purpose of as indicated in the ‘Data Analysis’ sub-section as sub-themes.

### Data collection

The data was collected from two different islands of Indonesia with diverse socio-economic conditions, Java and Sulawesi, to get diverse perspectives from hospitals that cater to patients with different socio-economic backgrounds. Java Island houses around 58.7 percent of Indonesia’s 270 million population while around 7.3 percent of the Indonesian population lives in Sulawesi Island [66]. Java Island is densely populated with 1,172 people living per square kilometer whereas only 105 people live per square kilometer on Sulawesi Island. Another major difference between these islands is in terms of the gross domestic product (GDP) each island generates for Indonesia. Java Island holds 58.7 percent of Indonesia’s GDP while Sulawesi holds only 6.7 percent of Indonesian GDP, indicating Java Island is economically more active and advanced than Sulawesi Island [66].

The units of analysis [64] for the research were twelve hospitals in Indonesia (six hospitals on each Island). The breakdown of the data collection methods in each hospital, the participants and their roles in the hospitals are presented in Table 1. As indicated in Table 1, we reached each of these hospitals to collect information related to the study. Once a hospital agreed to participate, its administration nominated employees they deemed suitable for providing insights into electronic medical record implementation and recordkeeping practices. This approach ensured the inclusion of diverse perspectives from staff members with relevant experience, while accommodating each hospital’s unique organizational context. Several hospitals engaged a greater number of hospital staff so we had to conduct both focus groups and interviews (online or offline) with different categories of hospital staff. On the other hand, some hospitals opted for engaging one key informant from the hospital to provide information about the hospital's recordkeeping practices in the context of EMR implementation. We also used online semi-structured questionnaires to reach some respondents because the health workers were busy serving patients. The link to the online semi-structured questionnaire was shared by the hospital research department to all health workers. We acknowledge that such distinction resulted in varied levels of information from different hospitals but it was based on the decision of our units of analysis (i.e., hospitals) regarding the representative information provided by the hospital staff engaged.

**Table 1:** Mapping of data sources of the research

Island	Hospital code	Data collection methods	Details
**Java**	A_Java (Government hospital, type A)	Interviews	Two (one IT personnel, one administrator)
		Questionnaire	Thirty-four (nine doctors, nineteen nurses, one pharmacist, five administrators)
	B_Java (Private hospital, type C)	Focus groups	Fourteen (one doctor, one nurse, four hospital managers, one laboratory analyst, two pharmacists, one radiographer, four administrators)
	C_Java (Teaching hospital, type B)	Focus groups	Twenty-two (seven doctors, ten nurses, two pharmacists, three radiographers)
	D_Java (Private Hospital, type B)	Focus groups	Thirty-two (thirteen doctors, eight nurses, one hospital manager, two laboratory analysts, three radiographers, four pharmacists, one administrator)
	E_Java (Government hospital, type C)	Focus group	Six (two doctors, one hospital manager, one IT personnel, two administrators)
	F_Java (Government hospital, type D)	Interviews	Two doctors
**Sulawesi**	A_Sulawesi (Government hospital, type A)	Interviews	Nine (five doctors, two nurses, one administrator, one IT personnel)
	B_Sulawesi (Government hospital, type B)	Interviews	Five (three doctors, one nurse, one administrator)
	C_Sulawesi (Government hospital, type C)	Interviews	Three (two nurses, one administrator)
	D_Sulawesi (Government hospital, type D)	Interviews	Three (two doctors, one administrator)
	E_Sulawesi (Government hospital, type C)	Interviews	Five (one doctor, one pharmacist, two administrators, one IT personnel)
		Focus groups	Eleven (two doctors, five nurses, two administrators, two IT personnel)
	F_Sulawesi (Private hospital, type C)	Interviews	Four (three doctors, one pharmacist)

Local languages were used during the interviews and focus group discussions to avoid misunderstanding. To ensure consistency in the data collection approach, the same two researchers collected the data from each hospital. The researchers moved to the next hospital upon data collection completion. Additional file 1 provides the questionnaire developed for the study to collect data and it was shared with the participants in advance (Additional file 1, pp. 1–2).

In Java, we conducted the interviews in six hospitals (two private hospitals, three government hospitals, and one teaching hospital). Each interview and focus group discussion lasted from 30 to 90 min. We recorded all interviews and focus group discussions and made anonymous transcripts. In Sulawesi, we conducted the interviews in six hospitals (two private hospitals and four government hospitals). Table 1 describes the demographic respondents involved in this study.

### Sampling strategy

The selection of hospitals in the research was purposefully sampled as purposive or purposeful sampling for qualitative research engaging information-rich units of analysis to gain an insight into the phenomenon [67]. Since our study is focused on hospitals, we primarily used our connections to get access to hospitals in Indonesia that can serve the purpose of the research to a level where the samples facilitate some degree of generalizability of findings. The protocol for interview and focus group was designed to understand recordkeeping practices of selected hospitals in light of EMR implementation so that different aspects of recordkeeping culture could also be understood. Following the protocol, we asked open-ended questions which were arranged in logical order and the same questions were asked to all interviewees while occasionally changing the order. As an example, the logical order followed in designing questions for hospitals begins with information about the participant(s), followed by information about the hospital, information about recordkeeping practices of the hospital in general and the practices around the implementation of EMR, and finally information on related enablers and barriers of implementing EMR.

### Data analysis

As thematic analysis assists in systematically analyzing qualitative data to identify themes or patterns that result in commonalties and relationships while explaining different principles [68], we used thematic analysis to analyze the qualitative data on recordkeeping practices related to EMR implementation of fourteen hospitals in Indonesia. While themes can be inductively generated from the raw information or deductively generated from theory and prior research [69], we used the deductive approach to generate themes by following the recordkeeping culture-related theoretical framework proposed by Oliver and Foscarini [14]. This resulted in three broad themes: A) foundations of electronic recordkeeping and information preservation in the hospitals, B) skills, knowledge and expertise of hospital employees relating to information management and recordkeeping, and C) hospital actions around recordkeeping in context of EMR implementation.

Sub-themes under each of the three themes were also generated by using deductive approach in light of the theoretical framework presented in Fig. 1. The sub-themes A1) Value accorded to records and respect for information as evidence, A2) Preferences for different communication media and formats and preferences for sharing information, and A3) The technological infrastructure in the island where the hospital is located were generated under theme one. Two sub-themes under the second theme, B1) Information-related competencies including information and digital literacy of hospital staff and B2) Staff awareness of societal, policy and organizational requirements relating to recordkeeping, were generated. Under the third theme, two sub-themes, C1) The information governance model in the hospital and C2) Recordkeeping systems and tools of the hospital, were generated.

The two researchers who collected the data analyzed the data first as in qualitative research, data analysis happens in conjunction with data collection. When data saturation in a hospital was reached, the researchers concluded the data collection in the particular hospital. To avoid bias, two other researchers analyzed the data and compared the findings. When there were disagreements, all four researchers took a look at the data again and engaged in discussions until the disagreements were resolved.

The research team’s backgrounds and positions influenced the study’s data collection, analysis, and interpretation. The team included members with diverse expertise in human-centered computing, computer science, and healthcare systems, providing both technical and contextual insights. However, familiarity with electronic medical record (EMR) systems and prior exposure to recordkeeping practices could have introduced implicit biases during data collection and thematic analysis. To mitigate this, as mentioned before, two researchers consistently conducted interviews and focus groups to maintain uniformity and multiple researchers independently analyzed the data to reduce subjective interpretation biases.

## Results of thematic analysis

In line with three major themes and seven sub-themes aligned with the recordkeeping culture-related theoretical framework proposed by Oliver and Foscarini [14], we found that despite valuing electronic records and having some institutional actions around EMR implementation, most of the hospitals in Indonesia have weak foundations around electronic recordkeeping and information preservation. Most of the hospitals do not also have adequate employees with skills, knowledge, expertise, and willingness relating to information management and electronic recordkeeping. This overall indicates a weak electronic recordkeeping culture among hospitals in Indonesia despite taking institutional actions largely due to the Indonesian government regulation requiring all healthcare facilities in Indonesia to implement EMR. However, some of the hospitals, especially in Java Island, demonstrated a strong electronic recordkeeping culture due to their proactive institutional actions around EMR implementation based on strong foundations around recordkeeping and information preservation while focusing on building skills, knowledge and expertise of their employees in relation to EMR management. In this section, we explain these findings related to different aspects of electronic recordkeeping culture.

### Foundations of electronic recordkeeping and information preservation in hospitals

While the first level of recordkeeping culture-related theoretical framework proposed by Oliver and Foscarini [14] is the foundational level, comprising of value accorded to records, information preferences and the regional technological infrastructure, we found that most of the hospitals in Indonesia covered in the research have weak foundations around recordkeeping and information preservation. This is reflected in the value they accord to records and respect for information as evidence, their preferences for different communication media and formats and preferences for sharing information, and the technological infrastructure in the island where the hospitals are operating. However, hospitals in Java Island have slightly better foundations than the hospitals in Sulawesi Island. Table 2 presents the summarized status of hospitals covered in the research around electronic recordkeeping and information preservation according to the three sub-themes related to the foundations of electronic recordkeeping and information preservation.

**Table 2:** Foundations of electronic recordkeeping and information preservation in hospitals

Hospital code	Foundations of electronic recordkeeping and information preservation in the hospitals		
	Value accorded to electronic records and respect for information as evidence	Preferences for different communication media and formats and preferences for sharing information	The technological infrastructure in the island where the hospital is located
**A_Java**	Value accorded to electronic record	Limited diversity in preferences	Inadequate resilience of technological infrastructure
**B_Java**			
**C_Java**		Limited diversity in source preference; Interest in preferring external information sharing	
**D_Java**		Limited diversity in preferences	
**E_Java**			
**F_Java**			
**A_Sulawesi**		Limited diversity in source preference; Interest in preferring external information sharing	
**B_Sulawesi**		Lack of information source preference and information sharing	
**C_Sulawesi**	Value accorded to electronic record partially		
**D_Sulawesi**			
**E_Sulawesi**	Value accorded to electronic record		
**F_Sulawesi**			

#### Value accorded to electronic records

The research data overwhelmingly indicates that in spite of minor scepticism, all the hospitals in Java and Sulawesi Islands of Indonesia covered in the research accord value to electronic medical records despite some staff of hospitals C_Sulawesi and D_Sulawesi are sceptic about the usefulness of EMR. The hospital employees consider that EMR allows them to systematically store, check, and visualize different data of patients, pharmaceutical products, and other hospital goods with ease that paper records are unable to offer. Electronic records can be accessed from different locations with easier searching efforts saving valuable time of employees and allowing greater flexibility to operate. This subsequently leads to better services for hospital patients in terms of quicker, longer and better services. In this regard, one of the pharmacists at C_Java hospital mentioned:

*In the inpatient pharmacy, we use hospital management information systems (SIMRS - Sistem Informasi Manajemen Rumah Sakit) every day. We see the patient’s treatment records in SIMRS when we want to process a prescription. For example, if we want to process a prescription, we must first see what the input or notes from the doctor. If we see a doctor prescribing drug A or drug B, we must first make sure whether it has been recorded in the medical record. It is to confirm whether the prescription is in accordance with the instructions of the doctor. We can cross-check first by looking at the integrated patient treatment record. So, what we use every day is a process for reliably offering medication to patients.*

Hospitals in Indonesia also value electronic records as they consider that such records are transparent and make the hospitals and medical professionals accountable due to the in-built performance-tracing mechanism. The importance of looking at patients’ medical histories has been mentioned by the hospital employees repeatedly as they opine that paper records can hardly facilitate that especially during emergency. Two administrators of E_Sulawesi hospital also highlighted the natural environment-friendliness of electronic record while valuing such records. The administrator indicated the paper waste generated for different purposes could be reduced to a great extent due to the use of electronic record.

Although we found most of the hospital staff accord value to electronic records, such accordance is both proactive and reactive. Several hospitals, mostly in Java Island like A_Java, B_Java, and C_Java, started to observe the positive benefits of electronic records for their operations and future growth. Hospital leadership was committed to allocate resources to adopt electronic recordkeeping systems and tools in the hospital as they observed the value of electronic record management. The regulations of the Indonesian Government requiring the hospitals to maintain electronic records were supportive for the proactive hospitals. For the reactive hospitals, the regulations pushed the hospitals to explore the usefulness of electronic medical record management and accord value to electronic records. Such differences between hospitals in terms of according value to electronic records have an impact on different electronic recordkeeping cultures of hospitals.

#### Information preferences

In relation to the preferences for different communication media and formats for getting information and for sharing information, it has been identified that most of the hospitals have limited or no diversity in their preferred media and formats for getting information and very limited thoughts about sharing the information externally. While it is understood that electronic text is considered as the main format for getting patient data by the hospitals, there is barely any thoughts about any other media and formats like digital photograph or email that they could utilize to strengthen EMR systems and practices. These hospitals consider that patients’ information can be obtained as electronic text through direct data entry when patients receive services in the hospitals. In this regard, other information media or formats for getting information from medical or consumer devices, imaging databases, or biorepositories could also be considered. However, the hospitals covered in our research demonstrated limited or no preference for these other media and formats. Although hospitals in Java have limited levels of diversity in their information preference, hospitals in Sulawesi have almost no preference for communication media and formats for getting information.

In terms of information sharing preferences, we also found that there is limited or no preference for sharing information beyond the hospital. There is a resistance to sharing certain information considering the sensitivity around personal information of patients. However, research participants did not discuss how information from patients could be aggregated, visualized and shared with policymakers to influence the health-related decision-making process continuously with real-time data without compromising patients’ privacy. We found that the use of EMR systems is mostly a daily activity without significant thoughts about its potential strategic use through information sharing by the hospitals. We found that the lack of preference for wider information sharing can be associated with the lack of time the health professionals have as they consider that information input is already taking away significant amount of their time. Doctors in several hospitals are reluctant to input the data in the system and engage someone else for data entry, disregarding patients’ privacy. So, there is a mixed scenario around the association of low-preference for information sharing and consideration of patients’ privacy. In this regard, one of the administrators at A_Java hospital mentioned:

*There were several doctors for whom I offered to activate the data in one health centre and there were also those who didn't want to because they thought that the data would reach the centre and suddenly at the centre it could go to hackers. So, resistance to sharing data with one another still exists because the data is indeed sensitive.*

Overall, we found that in terms of preference for diverse information sources and sharing information, most of the hospitals are reactive in nature and are not taking notable proactive steps to develop the health sector in Indonesia by having strategic information preferences associated with the EMR systems.

#### Regional technological infrastructure

As the successful implementation a national EMR system requires sound technological infrastructure, data collected from the hospitals indicate that the technological infrastructure is not resilient enough at the moment to fully support the EMR systems even if there is a directive from the government to implement the system. Among the technological infrastructures, consistent availability of Internet and stability of network have come up as major concerns. Lack of Internet and network outage often force the hospitals to adopt manual input of records and then input them again in the electronic system, increasing their overall workload. It is an important consideration to be made while mandating the use of EMR system without ensuring the soundness of technological infrastructure in a particular region. In this regard, one of the administrators at C_Sulawesi hospital mentioned:

*... just yesterday the network disappeared. The person who wants to make Badan Penyelenggara Jaminan Sosial Kesehatan (BPJS Kesehatan, or Social Security Agency on Health) guarantees needs a network. People who want to check BPJS cards will also need a network. If you are an emergency room patient, you must register first and if the network doesn't work, BPJS doesn’t work. That's why a group of people is waiting when it comes to registering for BPJS guarantees. It is bad if an emergency room patient arrives and wants to register but it takes a long time due to poor network connectivity.*

From our conversations with hospitals, we have found that available investment to develop the technological infrastructures of the hospitals and beyond from the part of the government is not prominent while the onus of implementing the EMR systems lies with the hospitals. Moreover, the need for a cybersecure technological infrastructure was indicated by some hospitals and we did not find evidence of cybersecure technological infrastructure or notable investment in this regard to run the EMR systems in hospitals. One of the doctors of A_Sulawesi hospital discussed the associated risks:

*I would like to elaborate [on] the risks related to electronic medical records. The first problem with the electronic medical record is security. From unauthorized access. … Then the second problem is related to backing up data as there have been several cases where the data was lost and cannot be accessed again due to a lack of quality backups. The third problem is hardware failure including the power outage. If these problems persist, there are medico-legal consequences for the doctors and health facilities in terms of failing to use the EMR system.*

Overall, it has been found that to develop a recordkeeping culture to support EMR adoption, hospitals have the major responsibility to allocate financial budget to develop internal technological infrastructures which may not be possible for all hospitals. Besides, such budget allocation is only adequate if the enabling regional technological infrastructure has adequate resilience by receiving budgetary support from the government.

### Recordkeeping skills, knowledge and expertise of hospital employees

While looking at the second level of the recordkeeping culture theoretical framework [14] where skills, knowledge, and expertise of people to manage records are explored within an organization, we found that overall, there is lack of skills, knowledge and expertise of hospital employees in terms of managing electronic records. Although most of them have a strong awareness of the regulatory requirements to adopt the EMR system in hospitals, most of them have low digital information-related competencies and little willingness to strengthen those competencies mostly due to their age. Most of them also have a lack of awareness about organizational requirements and almost none of them gave importance to societal requirements behind adopting the EMR system. However, hospitals in Java Island have better awareness about organization requirements than the hospitals in Sulawesi Island. Table 3 presents the summarised status of hospitals covered in the research around electronic recordkeeping and information preservation according to the two sub-themes related to the skills, knowledge and expertise of hospital employees in Indonesia.

**Table 3:** Recordkeeping skills, knowledge and expertise of hospital employees

Hospital code	Electronic recordkeeping skills, knowledge and expertise of hospital employees	
	Digital information-related competencies	Awareness of operating environment
**A_Java**	Low competency and low willingness	Strong awareness of regulatory and organizational requirements. No awareness of societal expectations
**B_Java**		
**C_Java**	High competency and high willingness	
**D_Java**	Low competency and moderate willingness	
**E_Java**	Low competency and low willingness	
**F_Java**		
**A_Sulawesi**		Strong awareness of regulatory requirements. Low awareness of organizational requirements and societal expectations
**B_Sulawesi**		Strong awareness of regulatory requirements. No awareness of organizational requirements and societal expectations
**C_Sulawesi**		
**D_Sulawesi**		Low awareness of regulatory requirements. No awareness of organizational requirements and societal expectations
**E_Sulawesi**		Strong awareness of regulatory requirements. No awareness of organizational requirements and societal expectations
**F_Sulawesi**	High competency and moderate willingness	

#### Information-related competencies

In terms of the hospital staff having digital information-related competencies to adopt and implement an EMR system, we found that most of the hospitals are struggling to have staff with such competencies. This is due to the fact that existing staff were well acquainted with the paper-based recordkeeping system of the hospitals and they earlier did not need to build competencies for electronic recordkeeping. Therefore, there is a general lack of unwillingness among those employees as they feel that the push by the government to implement the EMR system at the hospital compels them to acquire new competencies along with their primary skills as doctor, nurse or pharmacist. They feel that acquiring those competencies is increasing their workload as they need to go through training and spend time to get technical support while using the EMR system at the hospital. Such scenario favours younger staff as they get the opportunity to acquire digital skills as part of their education which the older staff did not get. This scenario also influences the hospitals to prefer recruiting younger staff when vacancies are open, which is unfavourable for older people looking for jobs without digital information-related competency. In this regard, one of the administrators of A_Java hospital highlighted:

*Due to directive from the Ministry of Health, there must be digital transformation in hospitals. Last year all hospitals had to implement SIMRS. However, most of the nurses who are too old can’t use SIMRS. Younger staff who are still 35 to 40 years old can use SIMRS. They are happy to accept the change.*

We have found that hospitals like C_Java and F_Sulawesi that have staff with better competency and willingness than most of the other hospitals emphasize staff electronic recordkeeping training and allocate resources to it. F_Sulawesi hospital has designed e-learning on the EMR system for its staff where staff have the opportunity of hands-on-training by managing the medical records of themselves and their family members. The hospital facilitates a process of continuous learning for the staff so that it becomes a habit of the staff. C_Java hospital invests in strengthening its information technology department so that the department not only supports the technical infrastructure development and maintenance but also assisst in building capacity of hospital staff. The hospital ensures that staff like doctors and nurses are recruited with skill to manage electronic records and get strong orientation when they start their employment. Overall, we found that the hospitals play an important role in terms of strategizing to build competency and willingness of staff in a systematic manner to use the EMR systems through redesigning their hiring and capacity building processes.

#### Awareness of operating environment

Awareness of requirements related to the operating environment is critical for staff of any organization to build the organizational recordkeeping culture. The operating environment-related requirements in this regard include organizational requirements, societal requirements, and regulatory requirements around the recordkeeping system, which in our research is the EMR systems for hospitals in Indonesia. Based on the data we found that while most of the hospital staff are aware about the regulatory requirements around adopting and implementing the EMR system in their hospitals due to the push by the Indonesian government, there is a lower level of awareness of organizational requirements to manage electronic records. In terms of considering societal requirements, hospital staff do not feel that there is any notable requirement or expectation that the society puts on them to implement the EMR system. Consequently, they do not have any urge to be aware about the societal requirements. In this regard, one of the hospital managers of B_Java hospital mentioned:

*For EMR and SIMRS, the government must be responsible because it makes policies. We just follow the government. If there is no electronic medical record data, the Ministry of Health will not allow permits and collaboration with BPJS.*

A similar opinion was also expressed by one of the administrators of E_Sulawesi hospital while highlighting the push from the Indonesian government to implement an EMR system:

*If any hospital is an accredited hospital but does not use electronic medical records, the government will review either its BJPS claims or accreditation and the collaboration with BPJS will be postponed. During the accreditation, the authority checks whether or not the digital recordkeeping is established. However, the E_Sulawesi Hospital demonstrated a strategic improvement plan during the previous accreditation, and they passed the process as a result of this plan, which included initiatives in digitized medical recordkeeping.*

From the discussion with hospitals, we found that the government’s requirement to implement an EMR system has been well communicated to the hospitals as staff are adequately aware about it. When some of the hospitals took the regulatory requirements seriously, they set up related organizational requirements accordingly. While some staff know those organizational requirements, they are also aware that those requirements are mostly emanated from the regulatory requirements. However, almost no hospital staff discussed whether or not there was any societal expectation on hospitals to implement an EMR system in hospitals. There were discussions around patients’ expectations around accessing their medical records with ease from different locations. But we did not find any evidence that the patients’ expectations were captured to design the EMR systems of hospitals while taking feedback on the systems from the patients. Consequently, the hospital staff are mostly unaware about the patients’ expectation to adopt an EMR system. Overall, we found that from an operating environment-related requirement awareness perspective for hospital staff, electronic recordkeeping in Indonesian hospitals by the staff is largely driven by the regulatory pressure of the government of Indonesia on the hospitals.

### Hospital actions around recordkeeping in context of EMR implementation

The third level of the recordkeeping culture theoretical framework [14] is focused on the actual recordkeeping practices based on the organizations’ foundational arrangements and staff engagement to facilitate the actual practices. As per the framework, therefore, we looked at the information governance model of the hospitals that facilitate the EMR adoption and implementation as well as the associated systems and tools used for such adoption and implementation. Overall, we found that most of the hospitals have an existing information governance model to facilitate the EMR adoption and implementation mostly based on learnings from the previous past paper-based information governance model. However, for several hospitals, the information governance model for EMR implementation is still evolving. This is also reflected in having EMR systems and tools in place since most of the hospitals have good to moderate level of EMR systems and tools usage. For several hospitals, such usage is still evolving. It has been found that due to the transition from paper-based records to electronic records, paper-based systems and tools are still relevant for most of the hospitals as they often adopt hybrid systems and tools. While comparing the hospitals’ actions around recordkeeping in the context of EMR implementation in Java Island and Sulawesi Island (presented in Table 4), we found that hospitals in Java Island are advanced in their actions in having a related information governance model and using associated EMR systems and tools in comparison to the hospitals in Sulawesi Island.

**Table 4:** Hospital actions around recordkeeping in the context of EMR implementation

Hospital code	Hospital actions around recordkeeping in the context of EMR implementation	
	Information governance model	Recordkeeping systems and tools
**A_Java**	Existing model to implement EMR	Available EMR system and tools with some hybridity
**B_Java**		
**C_Java**		Available EMR system and tools
**D_Java**		Moderately available EMR system and tools with some hybridity
**E_Java**	Evolving model to implement EMR	
**F_Java**		
**A_Sulawesi**	Existing model to implement EMR	Available EMR system and tools with some hybridity
**B_Sulawesi**		
**C_Sulawesi**	Evolving model to implement EMR	Evolving EMR system and tools with significant paper-based tool usage
**D_Sulawesi**		
**E_Sulawesi**		
**F_Sulawesi**	Existing model to implement EMR	Available EMR system and tools with some hybridity

#### Information governance model

In relation to the information governance model to adopt and implement an EMR system, we found that most of hospitals in Indonesia have an existing information governance model or the model is evolving for those which do not yet have an established model to implement an EMR system. This is largely due to the fact that information governance is a business-as-usual function of the hospitals and they are familiar with the paper-based recordkeeping of patients’ information and other operational recordkeeping practices as part of that governance. The presence of a previous governance model paved the way to upgrade a new information governance model for EMR management. However, we found that the information governance for EMR management is mainly focused on electronic recordkeeping and information technology governance by strengthening the IT department, and complying to the government regulations. We found that less importance is given to information security, data privacy and risk management, which are also part of information governance for adopting an EMR system. In this regard, one of the administrators of A_Java hospital mentioned:

*We have our own server and we also have a data center. We are currently engaging a database administrator. To develop SIMRS, the infrastructure is required first with the network and computers. Computers are procured every year. From a security perspective, every computer has an antivirus, and we also have a firewall on the network. We are really focusing on developing SIMRS because we are developing it on a large scale from 2020, especially for hundreds of nursing care data for accreditation.*

In relation to information governance during the transition period when hospitals are switching from paper-based records to electronic records, we also got insights about the transitional information governance model of some hospitals that have an evolving information governance model to implement the EMR systems. While some hospitals have plans around disposal of paper records, we also found that several of them do not have a clear transition plan. In this regard, one of the administrators of E_Sulawesi hospital highlighted:

*Some hospitals have no idea what the management does with the paper records once their importance expires. Currently, inpatient paperwork is stored in the medical records counter and separated by patient types, such as paediatrics, obstetrics, general patients, and newborn patients, including patient status, such as outpatient care and inpatient care.*

One of the IT personnel of the same hospital further mentioned:

*At our hospital, the current paper records will be destroyed after 5 years. The digital records will also be kept for 5 years. According to medical data regulations, records are kept for 5 years, except for informed consent forms with different retention requirements.*

Overall, our data indicates that while some hospitals are advanced in terms of information governance model to implement an EMR system, the scenario varies from hospital to hospital due to the varied focus provided by those hospitals on different components of information governance.

### Recordkeeping systems and tools

We found that most of the hospitals that have an existing information governance model to implement an EMR system also have necessary recordkeeping systems and tools. In the hospitals for which the information governance model is evolving to implement an EMR system, paper-based systems and tools are still relevant as electronic recordkeeping systems and tools are evolving for them. However, hybridity, the mix of both electronic and paper-based recordkeeping systems and tools, is also relevant for the hospitals that have available electronic recordkeeping systems and tools. This hybridity is due to the transition from paper-based recordkeeping to electronic recordkeeping as well as to the challenges associated with technological infrastructure and staff competency. In this connection, one of the doctors of D-Sulawesi hospital discussed:

*The first barrier is…. it’s a new thing. Because it’s new, it’s a bit confusing, especially if the signal is poor. The signal must be available; if there’s no connection, then automatically there’s no input. Besides, the availability of laptops is limited. If the availability of laptops is limited, automatically recording information electronically is a challenge and patients have to wait in line. So, we have been adjusting to the existing system and during the transition we are using paper-based records.*

The hospitals, which are advanced in using electronic recordkeeping systems and tools, are generating electronic reports for the patients and appreciative about SIMRS due to its communicative features. To be extra cautious, advanced hospitals are also provisioning additional backup systems in case data is lost in SIMRS. These hospitals also ensure manual backups of some information since some data is not in SIMRS and there are network drops. These hospitals provision more computers with additional data storage capacity and speed to handle large volume of data. Overall, we found that due to SIMRS, most of the hospitals are having existing electronic recordkeeping systems and tools or are on track to have such systems and tools despite facing some challenges to ensure necessary technological support. Paper-based recordkeeping systems and tools therefore maintain their relevance in the hospitals.

## Discussion

**Fig. 2:**
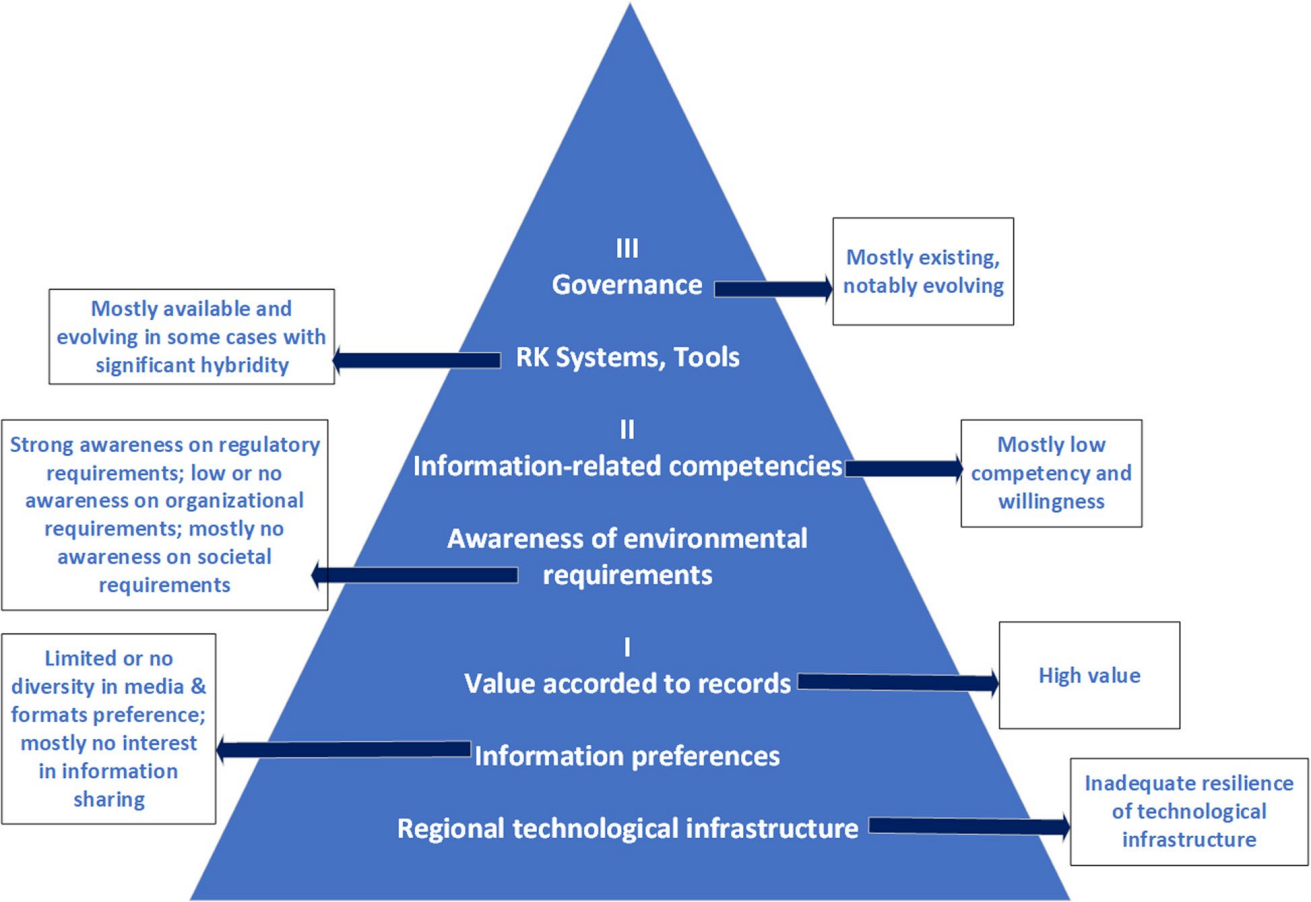
Electronic medical recordkeeping culture of hospitals in Indonesia

The culture of electronic recordkeeping within Indonesian hospitals, as revealed through the study of different parameters of recordkeeping culture, highlights both challenges and advancements in the adoption and integration of EMR. The findings suggest a wide variation in the foundational elements of recordkeeping, skills, and technological infrastructure across different hospitals, particularly when comparing institutions on Java Island and Sulawesi Island. Overall, while electronic records are valued for their efficiency and transparency, the overall recordkeeping culture in Indonesian hospitals remains weak, hindered by infrastructural limitations, staff competency, and strategic shortcomings. Based on the information culture framework proposed by Oliver and Foscarini [14], we capture the recordkeeping culture of hospitals of Indonesia in relation to EMR adoption and implementation in Fig. 2.

### Foundational elements of electronic recordkeeping in hospitals

The study indicates that most hospitals in Indonesia, especially those on Java and Sulawesi Islands, recognize the importance of EMR. EMR is seen as an efficient way to store, access, and share patient information, pharmaceutical data, and hospital operations with greater ease compared to traditional paper-based systems. Employees at various hospitals appreciate the ability to cross-check prescriptions, review patient histories, and offer reliable medication processing. Despite this positive view of EMR, the adoption is somewhat reactive for many hospitals, driven largely by the Indonesian government’s regulations, which mandate EMR implementation. Several hospitals in Java demonstrated proactive efforts by allocating resources and training staff, understanding the long-term operational benefits of electronic recordkeeping. In contrast, hospitals in Sulawesi showed less initiative, aligning their recordkeeping practices only to meet governmental mandates rather than a broader institutional strategy. This dichotomy mirrors findings from Harahap et al. [27], who emphasized the uneven readiness of healthcare facilities in Indonesia, especially in rural and economically disadvantaged areas, to adopt digital health technologies. Similarly, Sutrisni et al. [23] highlighted the influence of geographic and socioeconomic disparities on healthcare delivery in Indonesia, with urban hospitals often better equipped to adopt innovations than their rural counterparts.

The information provided by the hospital staff indicates that a significant obstacle to the effective implementation of EMR systems in Indonesia is the inadequacy of the technological infrastructure. Reliable internet access and stable networks are crucial for EMR, yet these are lacking, particularly in hospitals located on Sulawesi Island. Network outages frequently force hospitals to revert to manual systems, increasing the overall workload and slowing down processes. In addition to infrastructure challenges, hospitals demonstrated limited preferences for diversifying the media and formats used for acquiring information. Most hospitals relied exclusively on direct patient interactions for data input, with little consideration given to other potential media and formats such as those from imaging databases or biorepositories. This narrow view limits the potential scope and efficacy of EMR systems, as hospitals fail to fully utilize external information that could enhance healthcare quality. Information sharing is another challenge, with many hospitals hesitant to share data due to concerns about patient privacy and the potential for security breaches. The reluctance to share data externally highlights the need for more secure and resilient EMR systems to build confidence among healthcare professionals. This aligns with the findings of Derecho et al. [40] and Scholl et al. [43] on technology adoption in developing economies, which emphasized that robust IT infrastructure is a critical enabler for successful EMR implementation. Moreover, the study of Basani [26] on legal perspectives in Indonesia underscored the importance of secure and reliable technological systems to protect patient data while ensuring compliance with national regulations.

### Recordkeeping skills, knowledge and expertise of hospital employees

A critical aspect of fostering the development of an electronic recordkeeping culture is ensuring that employees have the necessary skills, knowledge, and expertise. However, the study reveals a general lack of these competencies among hospital staff. Most employees, particularly older ones, have little experience with digital information management and are often resistant to change. This finding is consistent with the research of Martinadewi and Gunawan [29], which identified staff unfamiliarity with EMR tools as a significant barrier to workflow integration in Indonesian hospitals. This is particularly evident in hospitals on Sulawesi Island, where many employees are comfortable with paper-based systems and view EMR as an additional burden rather than a tool for streamlining their work. However, hospitals on Java Island, have made more significant strides in addressing these skill gaps by investing in staff training and integrating the use of electronic records into everyday tasks. These proactive efforts by certain hospitals are crucial for developing an electronic recordkeeping culture, as they help employees adapt to new systems and integrate them into their work.

### Hospital actions around EMR implementation

While information governance is a vital aspect of recordkeeping culture in the context of EMR implementation, the study reveals that most hospitals in Indonesia are in a transitional phase, evolving from paper-based systems to hybrid models that combine both electronic and manual recordkeeping. Hospitals on Java Island, in particular, have made notable progress in establishing information governance models that support EMR adoption. However, many hospitals, especially in Sulawesi, are still developing their governance frameworks, often lacking clear policies for managing paper-to-digital transitions. While some hospitals have implemented EMR systems, they continue to rely on paper-based records due to infrastructure limitations and concerns about system reliability. This is in line with the study of Faida et al. [30], which demonstrated that leadership management and a positive organizational culture are essential for overcoming resistance to change and aligning hospital operations with digital health goals in Indonesia.

Finally, the ongoing use of paper records underscores the need for a more cohesive strategy to fully transition to electronic systems, ensuring that hospitals can securely store, access, and share patient data. While this transitional phase reflects the broader struggle of hospitals to balance traditional practices with modern requirements, the legal analysis of Basani [26] of electronic medical records in Indonesia highlighted the importance of clear policies and governance structures to manage this transition effectively.

### Regional disparities

Overall, the findings reveal stark regional disparities between Java and Sulawesi in EMR adoption, deeply rooted in technological, economic, and cultural contexts. Hospitals in Java benefit from robust technological infrastructure, higher levels of staff digital literacy, and proactive institutional strategies that foster a conducive electronic recordkeeping culture. Conversely, hospitals in Sulawesi often adopt EMRs reactively to comply with regulations, hampered by inadequate infrastructure, inconsistent internet connectivity, and limited staff training. These disparities underscore how the recordkeeping culture in Java is shaped by a forward-looking approach, leveraging resources to align technological advancements with staff competencies, whereas Sulawesi faces persistent challenges in building foundational readiness for EMRs. Additionally, the reluctance to share electronic records externally, particularly prevalent in Sulawesi, further impedes strategic data utilization and integration into broader healthcare frameworks. This finding is in line with the study of Rahman [21], which highlighted the challenge of urban–rural gaps in Indonesia the country’s geographical layout while offering the Universal Health Coverage (UHC) to citizens by the Government.

## Conclusion

The findings of this study on the electronic recordkeeping culture in Indonesian hospitals reveal a mixed landscape, with both challenges and advancements in the adoption and integration of Electronic Medical Records (EMR). Hospitals across the islands of Java and Sulawesi demonstrate varying levels of readiness and implementation, driven in part by governmental mandates. While many hospitals recognize the value of EMRs for their efficiency, transparency, and potential to improve patient care, the electronic recordkeeping culture remains generally underdeveloped, especially in terms of infrastructure, staff competency, and strategic integration. One of the key barriers identified is the lack of resilient technological infrastructure, especially in the more remote hospitals. This technological gap is a critical factor in the overall delay in the development of an electronic recordkeeping culture. Furthermore, the study highlights a significant skills gap among hospital staff from different generations in managing electronic records. This generational divide in digital literacy poses a challenge to the successful implementation of EMRs across all hospitals. Despite these challenges, there are examples of hospitals that have made notable progress in developing an electronic recordkeeping culture. These hospitals have invested in building the digital competencies of their staff and have integrated electronic records into their daily operations more effectively. However, even in these hospitals, hybrid systems that combine both paper and electronic records are still in use, highlighting the ongoing transitional phase.

Overall, we argue that while the adoption of EMRs in Indonesian hospitals is advancing, the success of this transition depends on addressing the infrastructural and competency-related challenges. A more cohesive strategy, with stronger technological support and targeted staff training, is needed to fully realize the potential of electronic recordkeeping in improving the quality of healthcare in Indonesia. In this regard, to address the challenge of poor Internet access, low-cost technological solutions like Starlink, a satellite-based Internet service by SpaceX, offer promising potential. By reducing the dependency on ground-based systems, Starlink ensures more reliable and secure connectivity, making it a viable option for enhancing digital inclusion efforts [70]. Partnerships with local governments or NGOs could further subsidize deployment costs and extend its reach to remote populations in Indonesia. Besides, to address the generational digital literacy gap, particularly among older healthcare professionals, targeted training programs, such as workshops or on-site support tailored to different learning paces, can help bridge this gap by providing hands-on experience and fostering confidence in using EMR systems. Additionally, offering incentives, such as continuing education credits or financial subsidies for completing training, can motivate participation and accelerate skill development. Peer mentorship programs, where tech-savvy younger staff support their colleagues, can also encourage intergenerational collaboration and enhance overall adoption rates.

Considering the regional differences as highlighted in our study, drawing from the experiences in Java, the Indonesian Government and the local governments in Sulawesi need to prioritize building a resilient technological infrastructure and fostering a collaborative culture for EMR adoption. Lessons from the initiatives of hospitals in Java, such as targeted digital literacy programs for healthcare staff, integration of EMRs into routine hospital workflows, and emphasis on data governance, can guide similar efforts for hospitals in Sulawesi. For example, leveraging e-learning platforms and peer mentorship programs, as seen in advanced Java hospitals, can accelerate digital competence among healthcare workers in Sulawesi. Furthermore, adopting a hybrid model that strategically transitions from paper-based to electronic records can address the immediate challenges of limited resources while setting the stage for a fully electronic system. Policymakers can support these efforts by channeling additional funding to underserved regions like Sulawesi and fostering inter-regional collaborations to share best practices.

The study highlights critical areas where policy interventions could significantly enhance EMR adoption in Indonesia. Firstly, national and regional governments need to prioritize investments in resilient technological infrastructure, especially in underserved regions like Sulawesi. For instance, policies incentivizing public–private partnerships to enhance internet connectivity and cybersecurity could address the infrastructural challenges that hinder EMR implementation. Secondly, the government could mandate and subsidize comprehensive digital literacy programs tailored to healthcare professionals, with a focus on intergenerational learning to bridge the digital divide. Finally, establishing standardized protocols for secure data sharing would not only alleviate privacy concerns but also promote the strategic use of EMR data for policymaking and public health improvements. A clear and enforceable framework for transitioning from paper-based to fully electronic systems, accompanied by financial and technical support, would ensure a smoother adoption process across hospitals.

This study provides valuable insights into the electronic recordkeeping culture in Indonesian hospitals, but it is not without limitations. While the findings highlight both advancements and challenges in the adoption of Electronic Medical Records (EMR) across different regions, certain constraints limit the generalizability of the results. First, the study focuses on hospitals in only two islands, Java and Sulawesi, which, although socio-economically diverse, may not fully represent the entire healthcare landscape of Indonesia, particularly in other regions with different healthcare infrastructures and cultural contexts. Expanding the research to include hospitals from additional islands could provide a more comprehensive understanding of the EMR implementation process.

Another limitation is the reliance on qualitative data from interviews and focus groups with hospital staff. While these methods provide deep insights into staff attitudes and experiences and rich contextual insights, they are inherently subjective and limits generalizability. To complement these findings, future research could incorporate quantitative measures, such as the number of EMR errors or time spent on recordkeeping tasks, to complement the qualitative findings and offer a more objective assessment of the impact of EMR adoption. Such metrics could provide a clearer picture of the impact of EMRs on hospital efficiency and patient care, offering a robust basis for scaling solutions across diverse healthcare settings. At that point, advanced qualitative data analysis tools such as NVivo could also be utilized for systematic coding and analysis as well as data visualization since the current study relied on manual data coding and analysis by the researchers. Moreover, due to the request of the hospitals, some discussions were conducted online, which may have posed challenges such as limited rapport and weaker group dynamics. While we attempted to overcome these challenges by establishing initial contacts with the staff prior to the discussions, and encouraging engagements during the discussions, online discussions are inherently more limited than in-person discussions.

Additionally, the study’s timeframe coincided with the initial phase of EMR implementation. Many hospitals were still transitioning from paper-based to electronic systems. A longitudinal study that follows the hospitals over a longer period would be valuable in assessing the sustained effects of EMR adoption and identifying whether the challenges observed, such as resistance to change among older staff or issues with technological infrastructure, are overcome as hospitals gain more experience with the system. Lastly, while this study focuses on the organizational and technological aspects of EMR adoption, this study does not fully explore the patient perspective on EMR adoption. Therefore, future research should investigate patients’ perceptions of EMR systems, as their views are crucial to shaping the recordkeeping culture of the hospitals. Understanding patients’ trust in data security, preferences for digital versus paper records, and their expectations for accessibility could offer valuable insights for optimizing EMR design and implementation. Additionally, exploring patient satisfaction with EMR-based care could illuminate broader cultural and operational shifts in healthcare recordkeeping in Indonesia.

In conclusion, while this research sheds light on the current state of electronic recordkeeping in Indonesian hospitals, future studies addressing these limitations would provide a more nuanced and complete picture of the challenges and opportunities for EMR systems in Indonesia’s healthcare sector. Moreover, the findings from this study resonate with challenges observed in other developing countries like Tanzania and Nigeria, where disparities in infrastructure and digital literacy are prevalent while adopting EMR [41, 44]. However, there are also strategies to be learnt from developing countries like India [42, 43], which implemented tiered digital health strategies, demonstrating the potential of scalable and resource-sensitive approaches in diverse healthcare settings. Therefore, drawing parallels between the experiences of Indonesia and other developing countries could help policymakers contextualize successful strategies and adapt them to the local socio-economic and cultural contexts across the developing world.

## Supplementary Information

Additional file 1.

## Data Availability

The datasets used and/or analyzed during the current study are available from the corresponding author on reasonable request.

## Abbreviations

EHR: Electronic health record
EMR: Electronic medical record
ISO: International organization for standardization
UHC: Universal health coverage
