# Supplementary material for: An exploratory study of electronic medical record implementation and recordkeeping culture: the case of hospitals in Indonesia
Source: BMC Health Serv Res. 2025 Feb 14;25:249. doi: 10.1186/s12913-025-12399-0 (PMC11827342; doi:10.1186/s12913-025-12399-0)
Supplement: Supplementary file 1 — Additional file 1. [file 12913_2025_12399_MOESM1_ESM.docx]

**Additional file 1: Questionnaire for Interview/FGD**

1. How familiar are you with EMR systems?
2. How often do you use an EMR system?
3. What are the current record-keeping practices in health facilities in Indonesia?
4. How do you observe the development of medical record-keeping practices, and what factors are contributing to the possible change in medical record-keeping practices from paper-based to electronic record-keeping?
5. What cultural challenges do healthcare providers typically face when transitioning to electronic record-keeping systems?
6. What are the main obstacles to the successful implementation of EMR in healthcare facilities in Indonesia?
7. How do you assess the impact of government initiatives in the implementation of EMR in all health facilities, on overall healthcare recording and delivery practices?
8. Are there significant geographic variations in EMR readiness and acceptance in different regions in Indonesia?
9. How do socio-economic factors contribute to or hinder the effective implementation and utilization of EMR in health facilities?
10. What are the critical technology needs that healthcare facilities in Indonesia must address when implementing EMR?
11. What training or education programs are most beneficial for healthcare workers in adapting and maximizing the use of EMR?
12. What are your expectations regarding the use of technology to manage medical records? What do you think patients expect regarding the use of technology to manage their medical records?
13. What policy changes or support will improve the success of EMR implementation?
14. How can the implementation of EMR in hospitals meet compliance from the Ministry of Health and BPJS Kesehatan?
15. With the non-fulfillment of the implementation of electronic medical records, hospitals can be reviewed for accreditation by the Ministry of Health or cannot make claims to BPJS Kesehatan?
